# Real-time monitoring of *Pseudomonas aeruginosa* biofilm formation on endotracheal tubes in vitro

**DOI:** 10.1186/s12866-018-1224-6

**Published:** 2018-08-14

**Authors:** Eva Pericolini, Bruna Colombari, Gianmarco Ferretti, Ramona Iseppi, Andrea Ardizzoni, Massimo Girardis, Arianna Sala, Samuele Peppoloni, Elisabetta Blasi

**Affiliations:** 1 Department of Surgical, Medical, Dental and Morphological Sciences with interest in Transplant, Oncological and Regenerative Medicine, University of Modena and Reggio Emilia, Modena, Italy; 2 Department of Life Sciences, University of Modena and Reggio Emilia, Modena, Italy

**Keywords:** *Pseudomonas aeruginosa*, Biofilm, Real-time monitoring, Endotracheal tube, Bioluminescence

## Abstract

**Background:**

*Pseudomonas aeruginosa* is an opportunistic bacterial pathogen responsible for both acute and chronic infections in humans. In particular, its ability to form biofilm, on biotic and abiotic surfaces, makes it particularly resistant to host’s immune defenses and current antibiotic therapies as well. Innovative antimicrobial materials, like hydrogel, silver salts or nanoparticles have been used to cover new generation catheters with promising results. Nevertheless, biofilm remains a major health problem. For instance, biofilm produced onto endotracheal tubes (ETT) of ventilated patients plays a relevant role in the onset of ventilation-associated pneumonia. Most of our knowledge on *Pseudomonas aeruginosa* biofilm derives from in vitro studies carried out on abiotic surfaces, such as polystyrene microplates or plastic materials used for ETT manufacturing. However, these approaches often provide underestimated results since other parameters, in addition to bacterial features (i.e. shape and material composition of ETT) might strongly influence biofilm formation.

**Results:**

We used an already established biofilm development assay on medically-relevant foreign devices (CVC catheters) by a stably transformed bioluminescent (BLI)-*Pseudomonas aeruginosa* strain, in order to follow up biofilm formation on ETT by bioluminescence detection. Our results demonstrated that it is possible: i) to monitor BLI-*Pseudomonas aeruginosa* biofilm development on ETT pieces in real-time, ii) to evaluate the three-dimensional structure of biofilm directly on ETT, iii) to assess metabolic behavior and the production of microbial virulence traits of bacteria embedded on ETT-biofilm.

**Conclusions:**

Overall, we were able to standardize a rapid and easy-to-perform in vitro model for real-time monitoring *Pseudomonas aeruginosa* biofilm formation directly onto ETT pieces, taking into account not only microbial factors, but also ETT shape and material. Our study provides a rapid method for future screening and validation of novel antimicrobial drugs as well as for the evaluation of novel biomaterials employed in the production of new classes of ETT.

**Electronic supplementary material:**

The online version of this article (10.1186/s12866-018-1224-6) contains supplementary material, which is available to authorized users.

## Background

It is known that microbial biofilm frequently forms on the surface of endotracheal tubes (ETT) implanted in patients receiving assisted ventilation [1–4]. In particular, biofilm occurs onto more than 90% of ETT within 7 days from insertion, since standard cleaning practices are not able to fully clear bacterial slime [5, 6]. As a consequence, ETT-associated biofilm increases the risk of upper respiratory tract infections, as well as device occlusion and wound infections, among other complications [7, 8]. Biofilm on ETT is recognized to be one of the main causes of ventilator-associated pneumonia (VAP) [9, 10]. The latter is commonly caused by the opportunistic pathogen *Pseudomonas aeruginosa* (*P. aeruginosa*) [11]*. P. aeruginosa* is a Gram negative bacterium that possesses several virulence tracts, some of which are cell-associated moieties (like flagella, pili, lectins, alginate/biofilm, lipopolysaccharide) while some others are secreted (namely proteases, hemolysins, cytotoxin, pyocyanin, siderophores, exotoxin A, exoenzyme S, exoenzyme U, etc.) [12]. Furthermore, *P. aeruginosa* is one of the greatest biofilm producers.

Soon after intubation, bacteria can adhere and multiply on the ETT surface to form biofilm. From there, entry of opportunistic pathogenic bacteria is facilitated so that the lower respiratory tract as well as the lung parenchyma are often involved [2, 11]. Notoriously, biofilm represents a complex and tightly adherent microbial community, embedded in an abundant matrix of hydrated extracellular polymeric substance (EPS), primarily composed of polysaccharides, proteins, nucleic acids and lipids [13]. From biofilm, microbial cells detach and easily get access to the lower airways through ventilator gas flow and aspiration [2]. Therefore, biofilm on ETT provides a persistent reservoir of pathogens likely responsible for VAP [3, 10, 11]. *P. aeruginosa* biofilm structure and stability are determined by at least three different polysaccharides, namely alginate, Pel and Psl [14, 15]. In particular, alginate is able to stabilize biofilm structure and to contribute to water retention and nutrients accumulation inside the matrix [16]. Another relevant component of *P. aeruginosa* biofilm is extracellular DNA (eDNA), which is known to play a key role in the onset and growth of *P. aeruginosa* biofilm, thanks to its ability to act as a cell to cell interconnection compound [13, 17, 18] and to be precious nutrient source for the embedded bacteria [19].

Once established, biofilm can resist to antibiotics and host immune response [20–23]. New chemical and mechanical approaches are currently under study to fight biofilm formation on ETT. The use of modified ETT (e.g. cuffed ETT and silver or other nanoparticle-coated ETT) have been shown to decrease the incidence of VAP in adults [24, 25]. Nevertheless, biofilm remains a serious health problem, requiring the development of efficacious preventive and therapeutic anti-biofilm approaches. In addition to bacterial factors, a largely underestimated feature, i.e. the combination of ETT shape and material composition, may strongly influence biofilm formation [26]. Indeed, most of our present knowledge on *P. aeruginosa* biofilm development onto ETT originates from in vitro studies on abiotic substrates, such as polystyrene wells or plastics per se, without taking into account other structural and three-dimensional parameters.

In the present work, by using a bioluminescent strain of *P. aeruginosa*, we describe a rapid and easy-to-perform in vitro system for real-time monitoring *P. aeruginosa* biofilm formation, directly onto ETT. Through this procedure, it was also possible to evaluate the biofilm three-dimensional structure, the metabolic behaviour and the expression of microbial virulence traits of bacteria embedded within ETT-biofilm.

Therefore, our innovative approach allows us to study biofilm formation directly onto medical devices, taking into account not only microbial factors but also the combination of ETT shape and material. In our hands, we believe that such model mimics much more closely the process of biofilm formation occurring in the clinical setting.

## Methods

### *Pseudomonas aeruginosa* strains

The following strains were used: the bioluminescent *P. aeruginosa* (P1242) (BLI-*Pseudomonas*) and *P. aeruginosa* PAO1 (ATCC 15692) (wild type (WT)-*Pseudomonas*). As detailed elsewhere, BLI-*Pseudomonas* expressed the luciferase gene and luciferase substrate under the control of a constitutive P1 integron promoter [27].

### *Pseudomonas aeruginosa* culture conditions and cell growth

Bacteria from − 80 °C glycerol stocks were initially seeded onto Tryptic Soy Agar (TSA) plates and incubated overnight at 37 °C; then, a fresh single colony was collected, inoculated into 10 ml of Tryptic Soy Broth (TSB) and cultured overnight at 37 °C. The culture was then washed and inoculated into fresh medium (TSB plus 2% sucrose) at a dilution 1:10. To create a bacterial growth curve, the optical density at 595 nm (OD_595_) was determined every 20 min using a spectrophotometer (Tecan Sunrise™). After each reading, 100 μl were collected and plated onto TSA in order to determine the number of Colony Forming Units (CFU). For all the experiments, starting bacterial suspension was adjusted at 5 × 10^4^ CFU/ml in TSB with 2% sucrose. To quantify the bioluminescence emission by BLI-*Pseudomonas* in the experimental groups, a calibration curve was generated in the microtiter plate. In particular, serial dilutions (starting from 1 × 10^7^/ml) of bacterial suspension in TSB with 2% sucrose were prepared and 100 μl of each dilution was seeded in a black transparent-well microtiter plate. The plate was immediately read by using Victor™ X Light 2030 Luminescence reader (Perkin Elmer).

### Endotracheal tube (ETT) pieces preparation

Twenty-four hours before each experiment, a sterile disposable paediatric endotracheal tube (ETT) (RUSCH 3.0 mm – 5.0 mm) was cut under a biological safety cabinet (to maintain sterile conditions), in pieces of 0.5 cm length, as previously described by Kucharikova S. et al., with minor modifications [28] (Additional file 1). A maximum of 4 ETT pieces were placed into 1.5 ml microcentrifuge tubes, covered with foetal bovine serum (FBS) and vortexed. Then, further 100–200 μl of FBS were added to each tube to completely cover all ETT pieces, before being incubated at 37 °C overnight in static conditions.

### Biofilm formation on ETT pieces

In order to allow biofilm formation on ETT pieces, 200 μl of overnight cultures of BLI-*Pseudomonas* or WT-*Pseudomonas* (both at 5 × 10^4^/ml) in TSB with 2% sucrose were seeded in 96 well-plates, containing 1 ETT piece/well, either in the presence or absence of gentamicin (4 μg/ml); the plates were then incubated at 37 °C for 90 min (adhesion period). After incubation, the ETT pieces were washed twice with PBS at room temperature (RT), transferred to new wells and incubated for 12, 24 or 48 h in fresh TSB with 2% sucrose with or without gentamicin (4 μg/ml) at 37 °C plus 5% CO_2_. Unless otherwise specified, every 24 h the culture medium was replaced with fresh medium with or without gentamicin. After 12, 24 or 48 h of incubation, ETT pieces were washed twice with PBS at RT and biofilms were analyzed both by our innovative method and by a standard methods (see below).

### Real-time monitoring of biofilm formation on ETT pieces by bioluminescence and CFU analysis

After biofilm formation on ETT pieces, as described above, the bioluminescence was measured by using the Victor™ X Light 2030 Luminescence reader (Perkin Elmer). Data is shown as Relative Luminescence Units (RLU).

In selected experiments, the ETT pieces, treated as above described, were washed twice with PBS at RT, transferred to a microcentrifuge tubes containing PBS (1 ml), sonicated for 15 min at 30,000 Hz in a water bath sonicator, then placed on ice, vortexed for 20 s and placed again on ice. Subsequently, 100 μl of each microbial suspension, appropriately diluted, were plated onto TSA and incubated for 48 h at 37 °C. After incubation, the CFU were counted and data was expressed as CFU/ml.

### Evaluation of biofilm formation on ETT pieces by transmitted-illumination confocal microscopy and crystal violet staining

After the 90 min of adhesion period (T0) and 24 and 48 h of exposure to BLI-*Pseudomonas* as above described, the ETT pieces were washed twice with PBS at RT and fixed with paraformaldehyde (PFA) for 30 min at 4 °C, washed twice with PBS and then analyzed by transmitted-illumination confocal microscopy Nikon LV 150 Confovis Microscope. In selected experiments, after the 12, 24 and 48 h of exposure to BLI-*Pseudomonas* as described above, the ETT pieces were washed twice with PBS at RT, transferred to new wells and then stained with 2% crystal violet (CV) to quantify total biofilm mass directly on the ETT. This was done according with Stepanović’s protocol, with minor modifications [29]. Prior to OD evaluation, the ETT pieces were removed from the wells and the OD were measured on the liquid phase only. The OD values were measured at 570 nm by using Sunrise™ spectrophotometer (Tecan).

### Analysis of alive/dead cells embedded in ETT biofilm

To discriminate alive/dead microbial cells directly within biofilm formed on ETT pieces, after 48 h of exposure to BLI-*Pseudomonas* in the presence or absence of gentamicin (4 μg/ml), as described above, the ETT pieces were washed twice with PBS at RT, transferred to new wells containing 200 μl of PBS and stained by using the “live/dead imaging kit” (Thermo Fisher Scientific) that employs propidium iodide (PI) to label dead cells and 5(6)-carboxyfluorescein diacetate (CFDA) to label alive cells. The staining protocol was conducted according to the Manufacturer’s instruction. After 15 min of incubation at 37 °C, the ETT pieces were washed twice with PBS and the fluorescence emission (PI excitation/emission: 528/645; CFDA excitation/emission: 485/528) was analysed by a multi-well fluorescence plate reader (Synergi HTX, BIOTEK). The results were expressed as relative fluorescence units (RFU). In parallel, microbial bacterial cultures (used as a control) were grown in planktonic form for 48 h with or without gentamicin, washed, centrifuged, resuspended in fresh medium and stained with the same kit.

### Quantification of eDNA and pyoverdine in cell-free supernatants from ETT biofilm

For the analysis of eDNA and pyoverdine in cell-free supernatants, after the adhesion period, ETT pieces were incubated for 24 or 48 h in TSB with 2% sucrose with or without gentamicin (4 μg/ml). Medium was never replaced during the incubation period, which was carried out at 37 °C plus 5% CO_2._ Then, cell-free culture supernatants were collected and tested for eDNA and pyoverdine content. In detail, the ETT pieces were removed and the supernatants were collected and centrifuged twice at 10,000 rpm for 15 min in order to remove the remaining bacteria. To exclude residual viable bacteria, 50 μl of the supernatants were seeded onto TSA plates and incubated for 48 h at 37 °C under aerobic conditions; no bacterial CFU on TSA plates were ever observed. To quantify eDNA concentration in the cell-free supernatants, 100 μl of supernatants were incubated with PI (1 μg/ml) for 15 min at 37 °C; then, fluorescence emission was quantified with a multi-well fluorescence plate reader (Synergi HTX, BIOTEK) (excitation/emission: 528/645).

In parallel, pyoverdine release was quantified in 100 μl of the same culture supernatants (excitation/emission: 360/460), according to a standard protocol [30]. The measured amounts of eDNA and pyoverdine were plotted as RFU mean of triplicate samples ± SEM.

 Moreover, microbial bacterial cultures (used as a control) were grown in planktonic form for 24 and 48 h with or without gentamicin (4 μg/ml) and cell-free supernatants were analysed for the presence of both eDNA and pyoverdine, as above described.

### Quantification of biofilm on coverslips

To produce biofilm on coverslips, 1 ml of overnight cultures of BLI-*Pseudomonas* (5 × 10^4^/ml) in TSB with 2% sucrose were seeded in 6-well-chamber slides containing coverslips in the presence or absence of gentamicin (4 μg/ml) and incubated for 24, 48 or 72 h at 37 °C plus 5% CO_2_. Every 24 h, the medium was replaced with fresh medium with or without gentamicin (4 μg/ml). After 24, 48 or 72 h of incubation, coverslips were washed twice with PBS at RT, air-dried, transferred to a microscope slide and analyzed, as previously described [31], with a Nikon Eclipse 90i imaging system equipped with Nomarski DIC optics (Nikon Instruments Inc. USA) (40X). Images were acquired by a DS-2Mv Nikon digital camera and analysed using the Nikon NISELEMENTS version D3.1 software. The thickness of the biofilm was measured and expressed in μm.

### Biofilm-related genes

After 24 h of exposure to BLI-*Pseudomonas* in the presence or absence of gentamicin (4 μg/ml), as described above, the ETT pieces were washed twice with PBS at RT, transferred in a microcentrifuge tubes containing PBS (1 ml), sonicated for 15 min at 30,000 Hz in a water bath sonicator, then placed on ice, pipetting up and down ten times. Then, 100 μl from each tube as well as of 100 μl of an overnight fresh culture of BLI-*Pseudomonas* (both 1 × 10^6^ cells) were used for DNA extraction by the rapid alkaline lysis method, as previously described [32]. The *lasR*-*lasI* quorum sensing (QS) genes [33], the *lasB, toxA* and the *algD* genes [34], as well as *proC* housekeeping gene [35], were determined by polymerase chain reaction (PCR) performed and analysed as described previously [36], using primers reported elsewhere [33–35].

### Statistical analyses

Statistical analysis was conducted using GraphPad Prism 7.0 software. Data depicted in Figures are the mean ± standard error (SEM) from replicate samples of 3–7 different experiments. Statistical analysis was carried out with either two-tailed Student’s t-test or one-way ANOVA with Bonferroni’s post-hoc test. Values of *p* < 0.05 were considered significant.

## Results

### Real-time monitoring of BLI-*Pseudomonas* biofilm on ETT

**Fig. 1:**
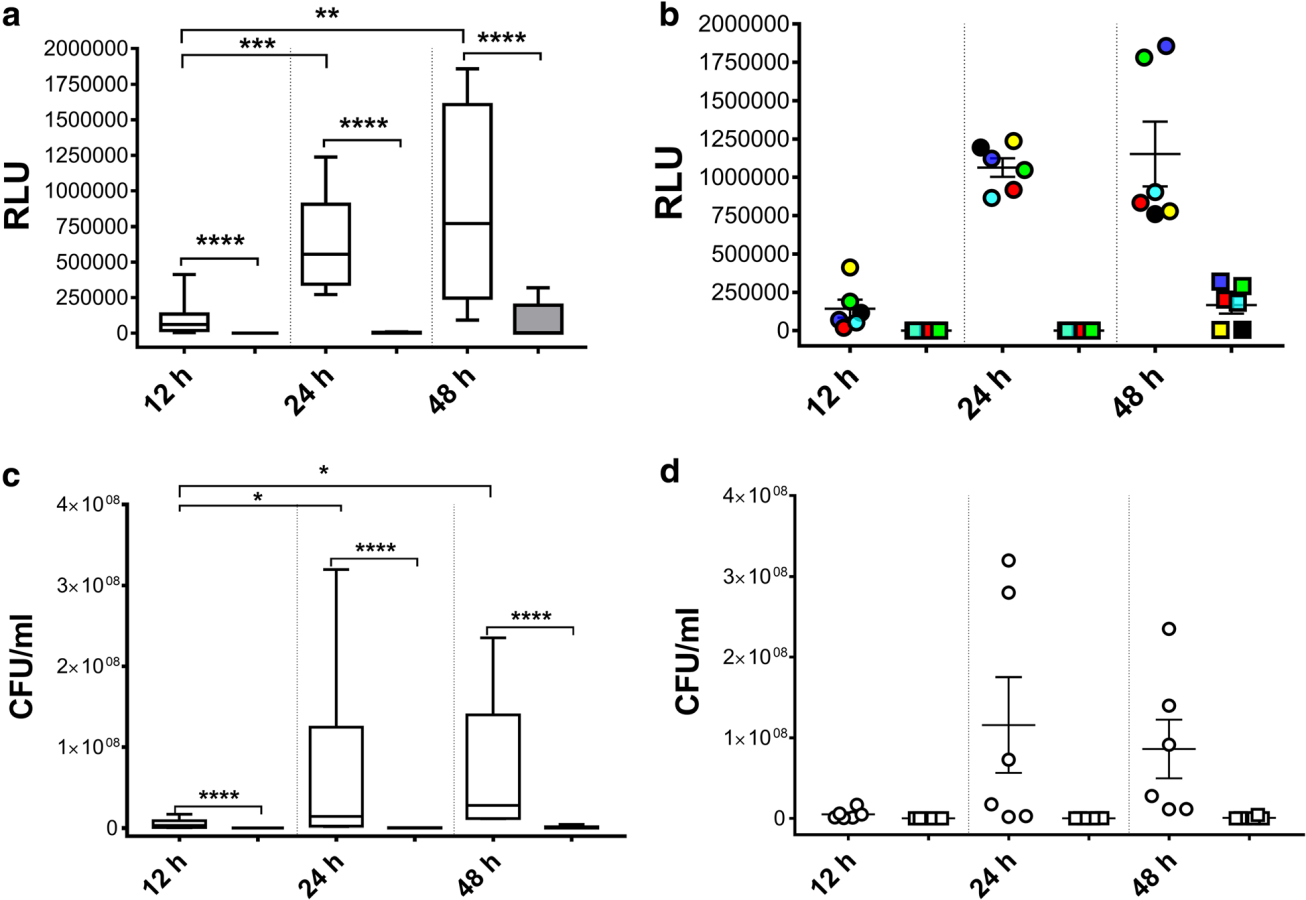
Real-time evaluation of biofilm formation on ETT pieces and CFU counts. Box plots of RLU (**a**) and of CFU/ml (**c**) of BLI-*Pseudomonas* biofilm on ETT pieces, untreated (white box plots) or treated (grey box plots) with gentamicin after 12, 24 or 48 h of incubation. Mean +/− SEM of RLU (**b**) or CFU/ml (**d**) analysis from 12 different ETT pieces. Circles or squares indicate the ETT pieces untreated or treated with gentamicin, respectively. Each ETT piece in panel **b** has a different colour: this allows us to follow the development of biofilm over time. Values of *p* < 0.05 (*), *p* < 0.01 (**), *p* < 0.001 (***) and *p* < 0.0001 (****) were considered significant

To investigate the possibility of real-time monitoring biofilm formation directly onto ETT pieces, we followed the bioluminescence emission from each single ETT piece exposed to BLI-*Pseudomonas* during a 12, 24 and 48 h incubation period. In parallel, the WT-*Pseudomonas* was used as negative control. Box plots in Fig. 1a show that the bioluminescence signal, expressed as RLU, significantly increased over the time. The treatment with gentamicin strongly affected biofilm formation on ETT pieces, at each time point tested. As expected, no bioluminescence signal was ever observed when using WT-*Pseudomonas* (data not shown). Figure 1b shows the results of representative experiments where groups of twelve samples (treated or not with gentamicin) were analysed in kinetics for bioluminescence emission. Each piece was marked with a different colour, so that it was possible to follow biofilm formation in each individual ETT piece over time. In selected experiments, we quantified the CFU (by standard method) from biofilm grown on ETT pieces, using the same experimental conditions. The results, depicted in Fig. 1c, showed that the number of CFU had a trend similar to that observed by bioluminescence analysis; nevertheless, the RLU results seemed to be more accurate in discriminating kinetic differences between the experimental groups. Figure 1d shows the kinetics of biofilm formation, assessed by means of CFU, on ETT pieces treated and untreated with gentamicin.

Using both tests (i.e. bioluminescence and CFU), no significant differences were observed between 24 and 48 h, suggesting that, in our experimental conditions, the maximum of biofilm biomass was achieved after 24 h. Gentamicin treatment significantly affected biofilm formation, both in terms of RLU and CFU (Fig. 1). Preliminary experiments, performed to construct a calibration curve, indicated that the analytical sensitivity of the bioluminescence signal from the BLI-*Pseudomonas* cells was more than 1000 bacterial cells (Additional file 2).

### Topographic analysis and evaluation of biofilm biomass on ETT

**Fig. 2:**
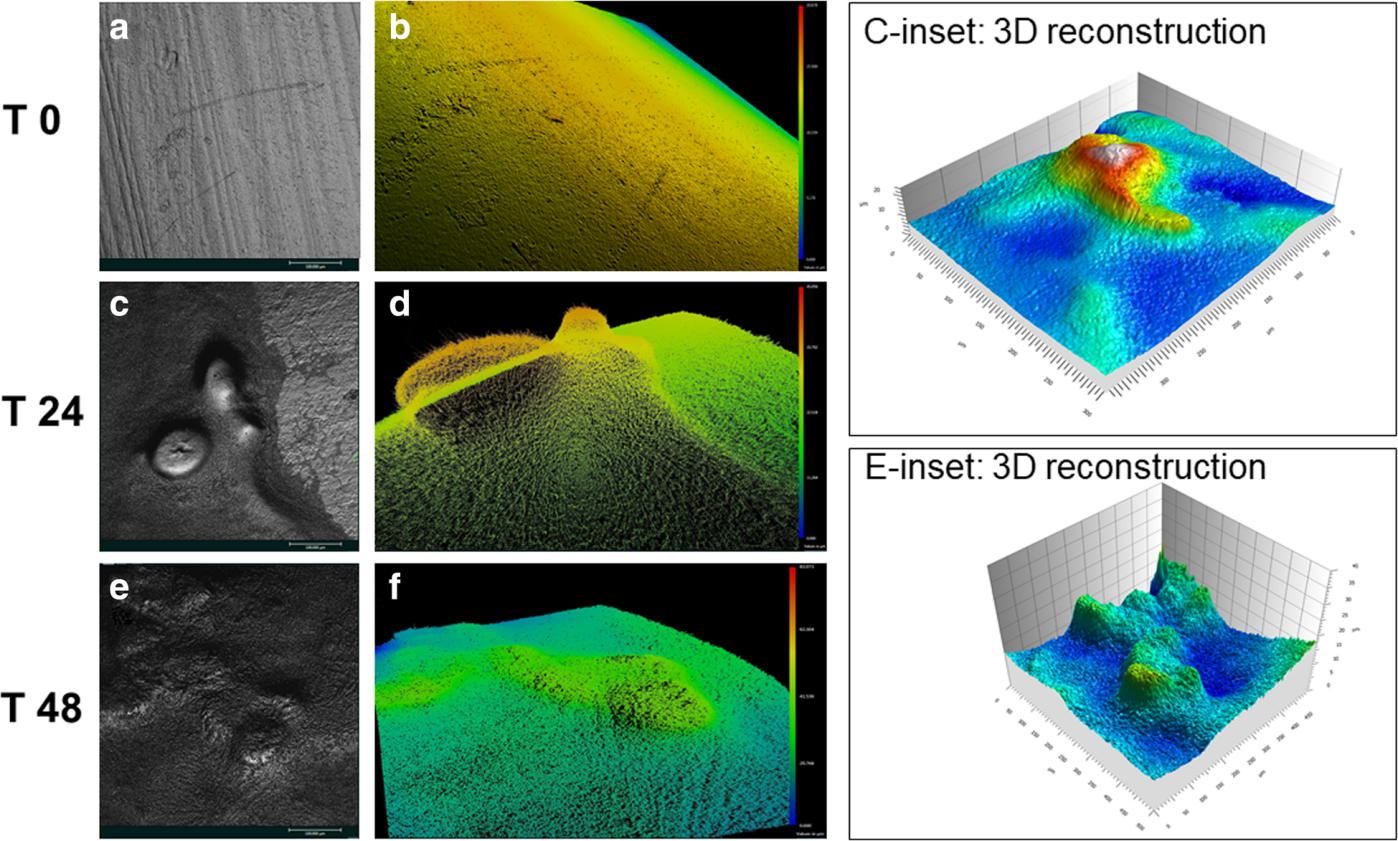
Confocal analysis of ETT biofilm. **a**, **c**, **e**: real black and white images acquired by transmitted-illumination confocal microscope of a 0, 24 and 48 h-old biofilm, respectively. These images result from the multiple acquisitions of several focal planes, which are ultimately combined. **b**, **d**, **f**: topography of the surface of a 0, 24 and 48 h-old biofilm, respectively, obtained by means of a false color scale. Specifically, the confocal microscope software allows us to see the differences in sample structure and thickness by assigning different colors to different areas. For each image, its own scale is given on the right side of the figure. The colored figures are tilted with respect to black and white correspondent images to highlight structure and thickness differences. C-inset and E-inset: 3D reconstruction of a 24 and 48 h-old biofilm, respectively, obtained by means of a mathematical linearization of the ETT surfaces. The images shown are representative of 2 independent experiments with the same pattern of results

To further characterize the biofilm structure on ETT, we performed a topographic analysis (3D mapping) of a 0, 24 and 48 h-old biofilm produced on ETT pieces, by transmitted-illumination confocal microscopy. The results show the absence of biofilm at 0 h (Fig. 2a and b). Moreover, Fig. 2c and e show the tridimensional architecture of the biofilm after 24 and 48 h of incubation with BLI-*Pseudomonas,* respectively. In addition, to better evaluate biofilm structure and thickness, a topographical reconstruction of the surface by means of a false color scale was carried out (Fig. 2b, d and f). Finally, a 3D reconstruction of 24 h and 48 h-old biofilm could be obtained by means of a mathematical linearization of the ETT surfaces, as shown in Fig. 2, C-inset and E-inset.

**Fig. 3:**
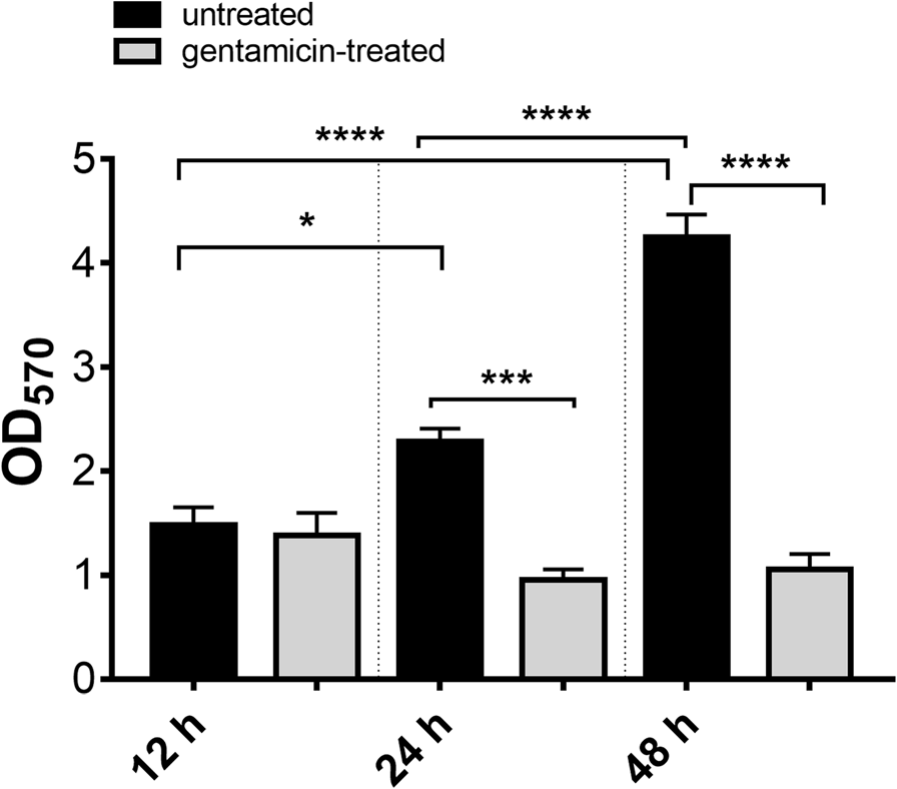
Crystal violet staining of ETT biofilm. Mean +/− SEM of biofilm mass analysed by CV staining (OD_570_). ETT pieces cultured for 12, 24 and 48 h with BLI-*Pseudomonas* in the presence (grey columns) or absence (black columns) of gentamicin were assessed. Values of *p* < 0.05 (*), *p* < 0.001 (***) and *p* < 0.0001 (****) were considered significant

The biofilm on coverslips was already detectable at 24 h and it kept on growing significantly up to 72 h of culture, when it reached 65 μm of thickness. No biofilm was produced on gentamicin-treated coverslip samples (Additional file 3). Crystal violet staining of 12, 24 and 48 h-old biofilm, directly on ETT pieces, showed a significant increase of biofilm mass over time. As expected, treatment with gentamicin significantly affected biofilm formation, being this effect particularly evident after 24 and 48 h (Fig. 3).

### Evaluation of both living and dead cells embedded in ETT biofilm

**Fig. 4:**
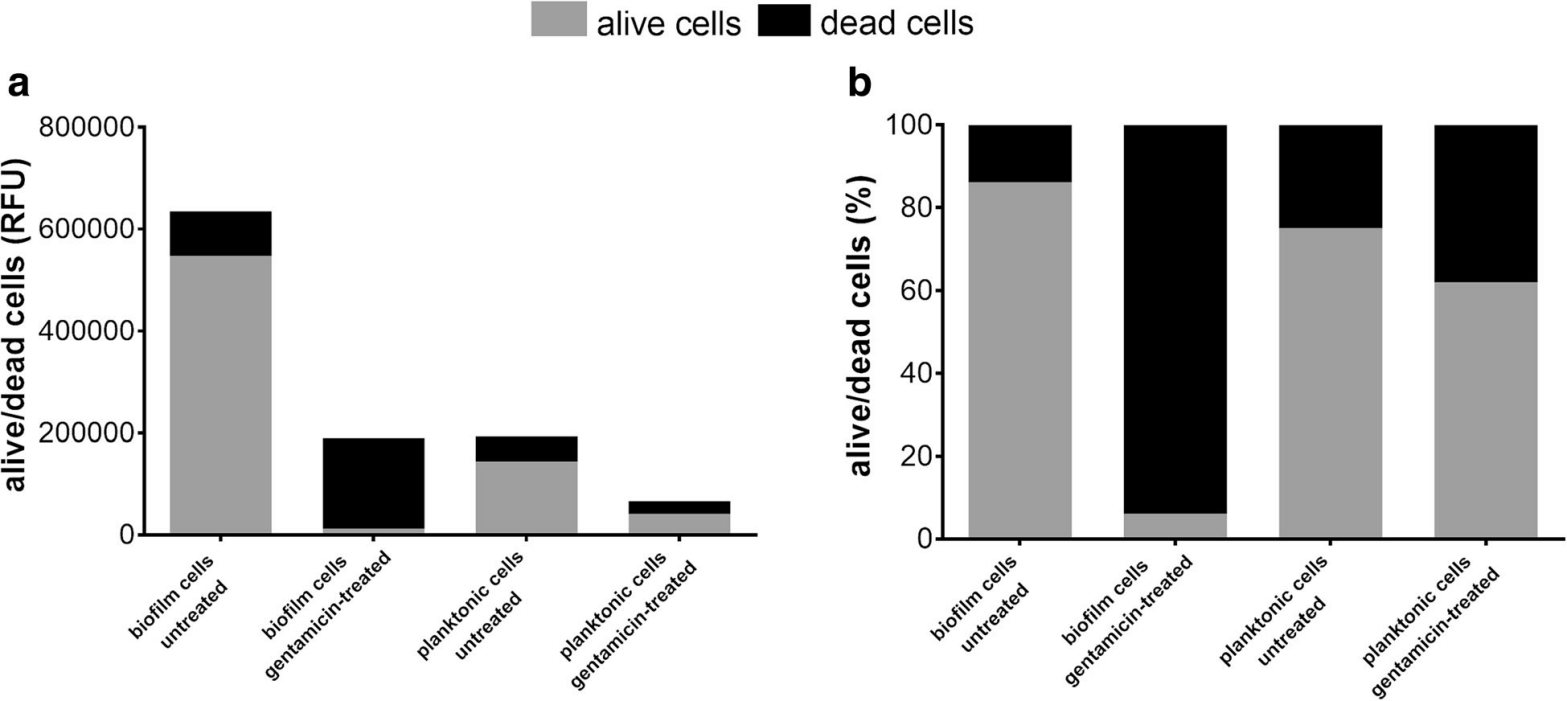
Quantification of alive/dead cells embedded in ETT biofilm. Relative Fluorescence Units (RFU) mean (**a**) and percentage (**b**) of alive (grey) and dead (black) cells into ETT 48 h-old BLI-*Pseudomonas* biofilm and planktonic cultures, treated or not with gentamicin. The data shown are representative of three independent experiments, which provided similar pattern of results

In order to demonstrate the versatility of our model, we investigated the metabolic changes in biofilm following gentamicin treatment, as a prototype of response to different therapies directly onto ETT. In particular, ETT pieces were incubated for 48 h with BLI-*Pseudomonas* either in the presence or in absence of gentamicin, and then stained with PI and CFDA to discriminate live from dead cells. As shown in Fig. 4, the addition of gentamicin strongly reduced the number of metabolically active and viable bacteria embedded in the polymer matrix of biofilm, as assessed by RFU (panel a) and percentage of alive/dead cells ratio (panel b), respectively. According to the evidence that the eDNA released from dead cells remains embedded within biofilm matrix [17], the signal of gentamicin-treated biofilm dead cells is much higher than the signal of gentamicin-treated planktonic dead cells.

### Evaluation of *P. aeruginosa* virulence traits in ETT biofilm

**Fig. 5:**
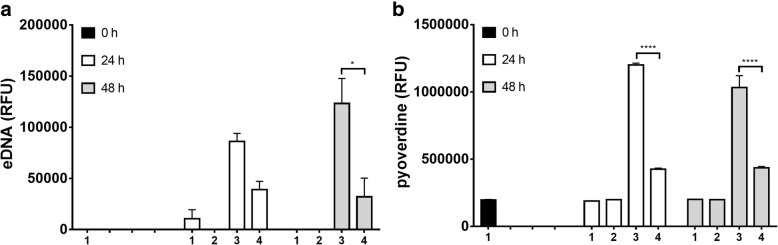
eDNA and pyoverdine determination in cell-free supernatants from ETT biofilm RFU mean +/− SEM of eDNA (**a**) and pyoverdine (**b**) production in cell-free supernatants of BLI-*Pseudomonas* biofilm on ETT pieces and planktonic cultures, untreated or treated with gentamicin, after 0, 24 or 48 h of incubation. 1: untreated planktonic cells 2: gentamicin-treated planktonic cells 3: untreated biofilm 4: gentamicin-treated biofilm ***p < 0.001 and *p < 0.05; gentamicin-treated samples *vs* untreated samples

It has been widely demonstrated that eDNA is a relevant component of *P. aeruginosa* biofilm, essential for the onset and stability of this sessile community [17, 18]. Hence, we analyzed eDNA directly on cell-free supernatants of ETT exposed to BLI-*Pseudomonas.* As shown in Fig. 5a, a consistent production of eDNA was observed 24 and 48 h later; moreover, as expected, eDNA production was significantly impaired following the gentamicin treatment. A minimal release of eDNA could be detected only from 24 h-old planktonic cultures.

In our model, we also analyzed pyoverdine release. Indeed, pyoverdine is a key pigment for in vivo iron gathering and virulence expression by *P. aeruginosa* [37]*,* and thus it contributes to promote the formation of biofilm [38]. Our results showed that significant levels of pyoverdine were released from 24 and 48 h-old BLI-*Pseudomonas* ETT biofilm. As expected, gentamicin treatment markedly reduced pyoverdine release (Fig. 5b). A basal level of pyoverdine release was detected from planktonic cultures at all testing time points, irrespective of gentamicin presence.

By analyzing some key genes involved in *P. aeruginosa* biofilm formation (such as *lasR* and *lasI*, *algD, lasB* and *toxA*) we found that they were all present in ETT-formed biofilm as well as in planktonic cells (gentamicin-treated biofilm served as negative control) and likely expressed in both. *proC* housekeeping gene was used as internal control (not shown) (Additional file 4).

## Discussion

Despite the numerous improvements in management of health-care associated infections, bacterial biofilm, often associated with the use of indwelling medical devices, remains a major concern. Indeed, once organized in a biofilm, microorganisms survive, in spite of host immune responses and antibiotic treatment. Biofilm formation is a multifactorial phenomenon and it is likely related to the production of a resistant EPS coating, to the reduction of microbial metabolism within the biofilm, and to the capacity of bacteria to share important drug-resistance genes [39]. Among numerous devices, ETT are especially susceptible to biofilm formation. They easily undergo microbial colonization and biofilm formation on their own surface, because of the concomitant skin/mucosa barrier breakdown as well as ETT exposure to respiratory mucus and blood that may further facilitate bacterial binding to such abiotic surface [7, 40, 41]. Finally, biofilm formation on ETT is widely feared since it provides a hidden bacterial reservoir that may delocalize and cause VAP among mechanically ventilated patients [10].

Most of our current knowledge of *P. aeruginosa* biofilm formation on medical devices, such as ETT, derives from in vitro studies on polystyrene or rough plastic sections [30, 42, 43]. Such models suffer from a relevant limitation, i.e. an underestimation of the role played by both device shape and material of ETT combined. These features, in addition to bacterial factors, may affect biofilm formation in vivo. For this reason, here, we describe a rapid and easy-to-perform in vitro method, which allows for a real-time evaluation of all the phases of *P. aeruginosa* biofilm formation onto a medical device. This method is designed to mimic, as close as possible, the real-life conditions, where the combination of microbial virulence factors and device shape and material all play a crucial role in biofilm formation.

On a previous work, Kucharikova and colleagues [28], have monitored real-time biofilm formation onto central venous catheter (CVC) pieces, using a well-established engineered strain of bioluminescent *C. albicans* (gLUC59) [44]. Starting from these data, here we have used an engineered strain of bioluminescent *P. aeruginosa* (BLI-*Pseudomonas*) to follow up in real-time biofilm formation on ETT pieces. Although the two models are conceptually quite similar, the BLI-*Pseudomonas* employed in this study offers a substantial advantage: unlike bioluminescent *C. albicans*, which needs the exogenous administration of the specific substrate (coelenterazine) to emit a quantifiable luminescence [44], BLI-*Pseudomonas* can be used and assessed as it is. BLI-*Pseudomonas* bears and constitutively expresses both the gene and the substrate necessary for the bioluminescence emission, as a results of the introduction of a specific *lux* operon [27, 45]. This peculiarity renders the system rapid and ready-to-use. The possibility to read the bioluminescence signal without any additional step simplifies the procedures and improves both precision and reproducibility. Moreover, the system allows multiple measurements in the same sample, thus allowing kinetic studies where manipulations and errors are minimized. Another advantage of our bioluminescence-based assays is that the read out of each experiment is not dependent on the operator’ skills, further strengthening the validity of this model. Although our study has been carried out only in vitro, future studies will focus on real-time monitoring of biofilm formation onto ETT pieces subcutaneously implanted in mice, similarly to what has been already done by Kucharikova and colleagues with CVC pieces [28].

In line with other bioluminescence analysis systems [28, 31, 46], here we show that quantification of bioluminescence by RLU provides data in line with those obtained by conventional techniques for biofilm quantification. When compared to techniques such as CFU and CV determination, our method requires shorter processing times and it provides more accurate data for discriminating kinetic differences among different experimental groups. Moreover, morphological analysis of the ETT-associated biofilm has been evaluated by confocal microscopy. Finally, quantification of viable cells can be easily performed as well. Indeed, by this approach we have been able to easily discriminate between living and dead cells embedded in the ETT biofilm, therefore assessing the viability of biofilm-embedded bacteria, in the presence and in the absence of gentamicin. Also, the expression of *P. aeruginosa* virulence traits (like eDNA and pyoverdine production as well as quorum sensing genes expression levels) have been directly measurable in ETT-structured biofilm. To our knowledge, this in vitro model mimics as close as possible what may happen in an in vivo situation, such as in the ventilator-assisted patients.

## Conclusions

Overall, we have described an original experimental model that, by providing a dynamic information on the development of a sessile microbial community onto medical devices, may find a rapid and practical application. Studies on the effectiveness of both new antimicrobial drugs as well as novel biomaterials, useful for designing and manufacturing innovative medical devices, may strongly benefit from such method. Therefore, the present model may find successful application not only in vitro but also in clinical studies.

## Additional files

Additional file 1: Preparation of paediatric ETT pieces. A sterile disposable paediatric endotracheal tube (ETT) (RUSCH 3.0 mm – 5.0 mm) was cut in pieces of 0.5 cm length, using a biological safety cabinet to ensure sterile conditions. (TIF 4704 kb)

Additional file 2: Calibration curve of BLI-*Pseudomonas* bioluminescence detection. Increasing numbers of BLI-*Pseudomonas* cells grown in TSB with 2% sucrose at 37 °C were analysed for bioluminescence emission by using luminometer. (PDF 80 kb)

Additional file 3: Evaluation of BLI-*Pseudomonas* biofilm on coverslips. The data represent the mean +/− SEM of BLI-*Pseudomonas* biofilm thickness (expressed in μm) on five different fields of each coverslip, untreated (black columns) or treated (grey columns) with gentamicin, after 24, 48 or 72 h of incubation. ***p < 0.001; 24 h-biofilm *vs* 72 h-biofilm ****p < 0.0001; gentamicin-treated biofilm *vs* untreated biofilm. (TIF 1096 kb)

Additional file 4: Analysis of biofilm-related genes. BLI-*Pseudomonas* quorum sensing genes (*lasR/lasI*, *lasB*, *toxA*, *algD)* in a 24 h-old biofilm formed on ETT pieces and in the planktonic cells are shown. Lanes show the bands of each gene, according to the length of fragments expected, detected by gel analysis: *lasR*: 725 bp; *lasI:* 605 bp; *lasB* 300 bp, *toxA* 352 bp and *algD* 1310 bp (*L* = ladder). Gentamicin-treated biofilm served as control. (PDF 50 kb)

## Abbreviations

ANOVA: Analysis of variance
ATCC: American type culture collection
BLI: Bioluminescence
CFDA: 5(6)-carboxyfluorescein diacetate
CFU: Colony forming units
CV: Crystal violet
CVC: Central venous catheters
DIC: Differential interference contrast
eDNA: Extracellular DNA
EPS: Extracellular polymeric substance
ETT: Endotracheal tubes
FBS: Fetal bovine serum
OD: Optical density
PBS: Phosphate-buffered saline
PCR: Polymerase chain reaction
PFA: Paraformaldehyde
PI: Propidium iodide
QS: Quorum sensing
RFU: Relative fluorescence units
RLU: Relative luminescence units
RT: Room temperature
SEM: Standard error of the mean
TSA: Tryptic soy agar
TSB: Tryptic soy broth
VAP: Ventilator-associated pneumonia
WT: Wild type
