# Supplementary figures and images for: Real-time monitoring of Pseudomonas aeruginosa biofilm formation on endotracheal tubes in vitro
Source: BMC Microbiol. 2018 Aug 14;18:84. doi: 10.1186/s12866-018-1224-6 (PMC6092828; doi:10.1186/s12866-018-1224-6)

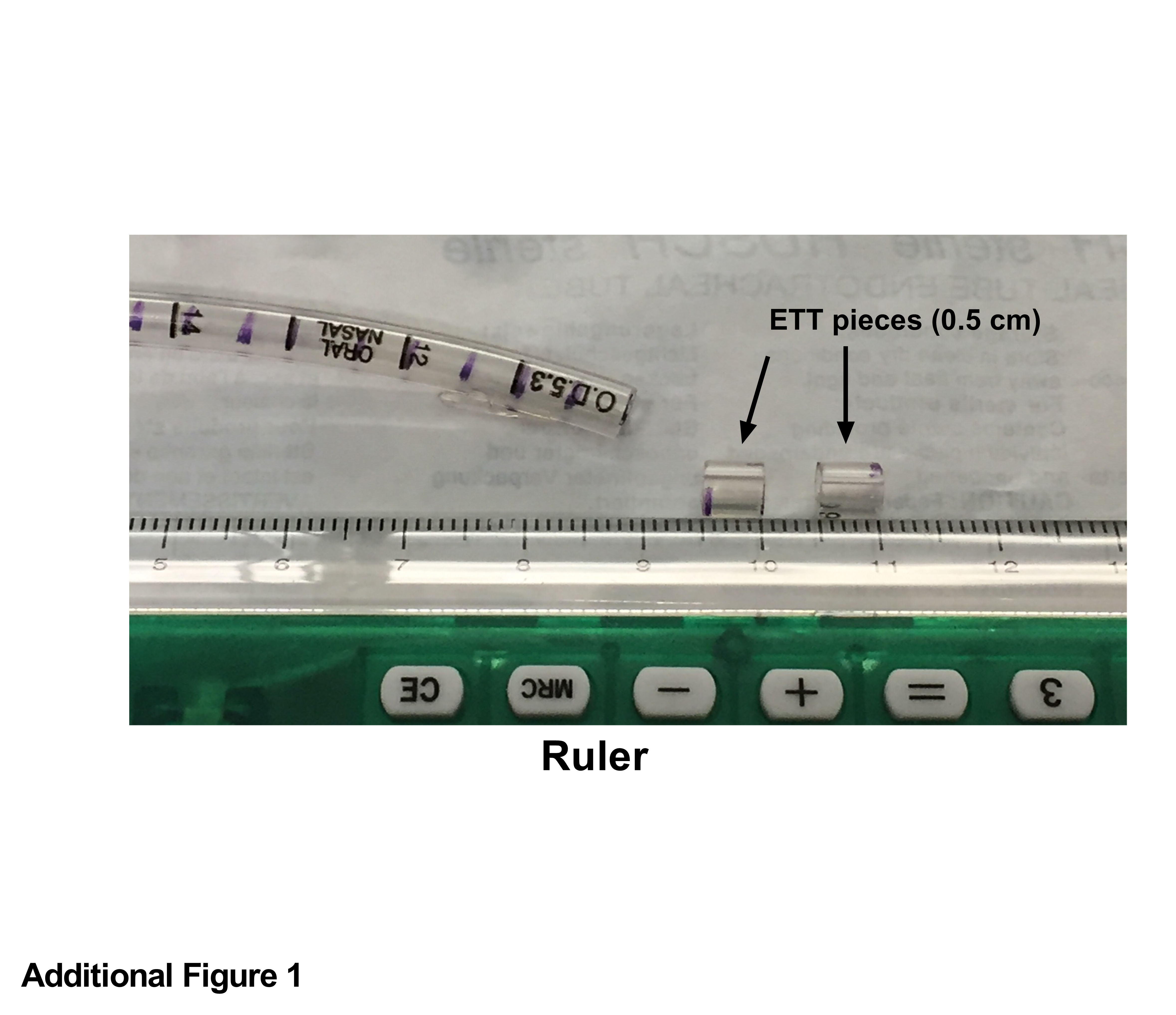

Supplement: Supplementary file 1 — Preparation of paediatric ETT pieces. A sterile disposable paediatric endotracheal tube (ETT) (RUSCH 3.0 mm – 5.0 mm) was cut in pieces of 0.5 cm length, using a biological safety cabinet to ensure sterile conditions. (TIF 4704 kb) [file 12866_2018_1224_MOESM1_ESM.tif]

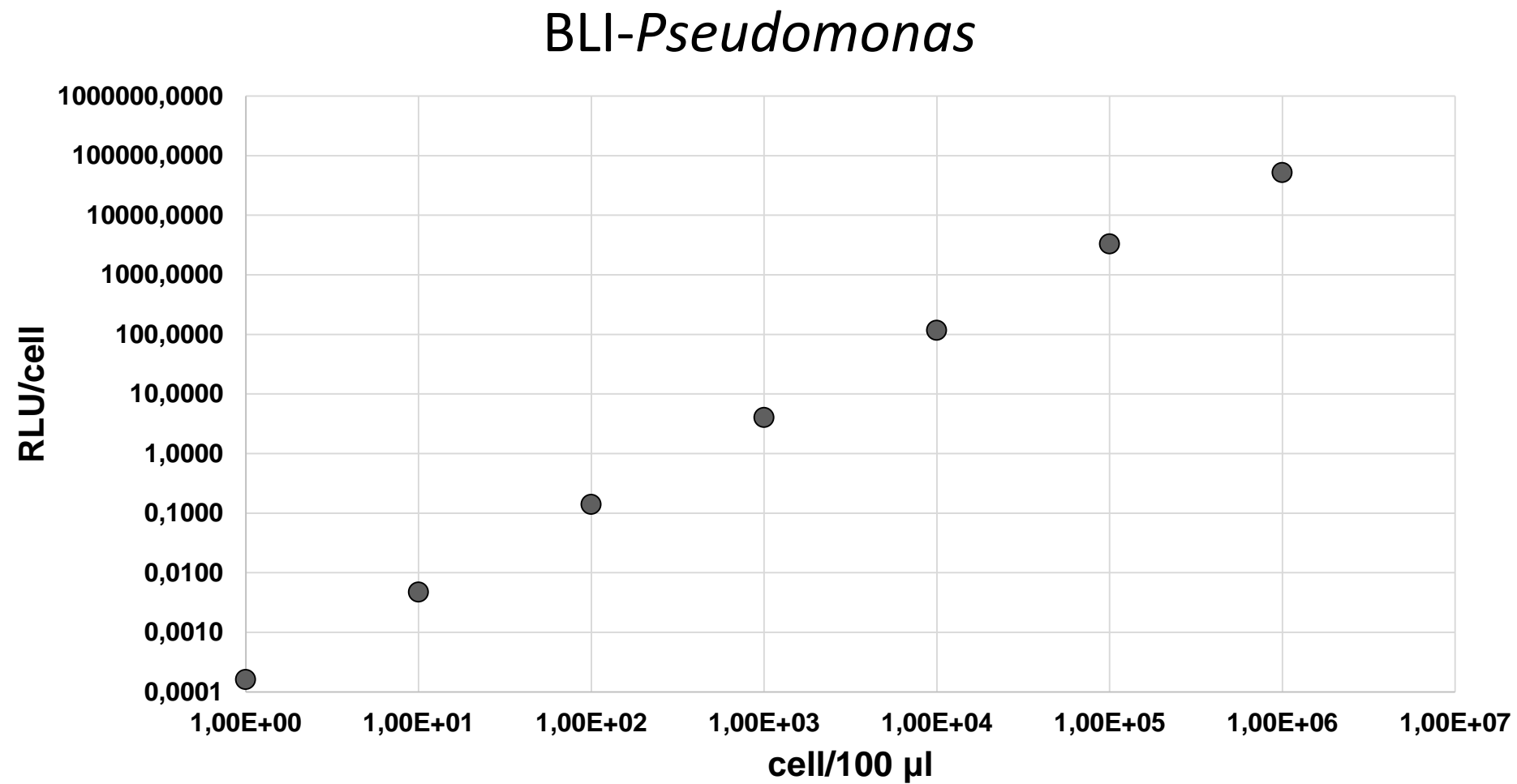

Additional figure 2

Supplement: Supplementary file 2 — Calibration curve of BLI-Pseudomonas bioluminescence detection. Increasing numbers of BLI-Pseudomonas cells grown in TSB with 2% sucrose at 37 °C were analysed for bioluminescence emission by using luminometer. (PDF 80 kb) [file 12866_2018_1224_MOESM2_ESM.pdf]

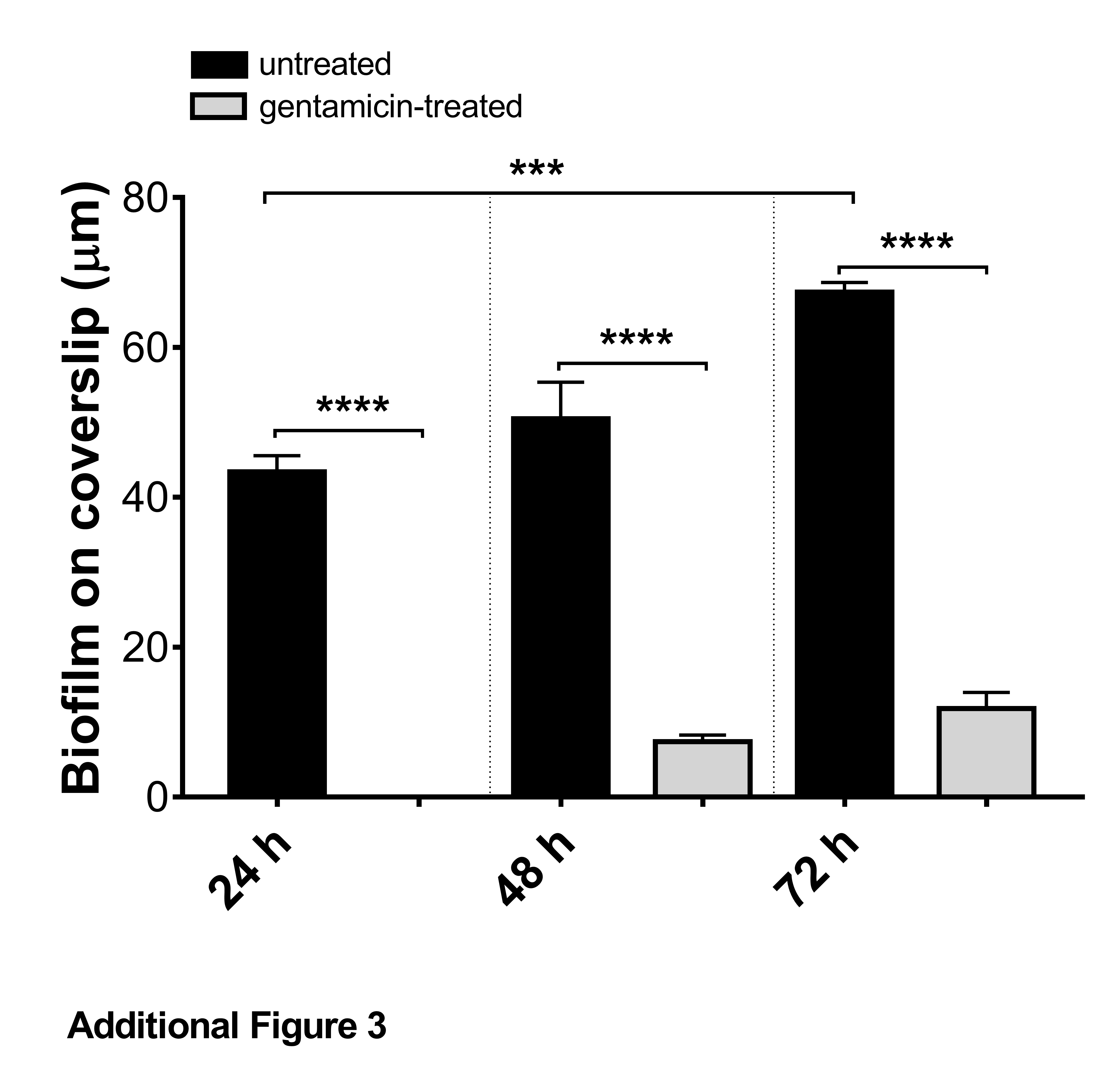

Supplement: Supplementary file 3 — Evaluation of BLI-Pseudomonas biofilm on coverslips. The data represent the mean +/− SEM of BLI-Pseudomonas biofilm thickness (expressed in μm) on five different fields of each coverslip, untreated (black columns) or treated (grey columns) with gentamicin, after 24, 48 or 72 h of incubation. ***p < 0.001; 24 h-biofilm vs 72 h-biofilm ****p < 0.0001; gentamicin-treated biofilm vs untreated biofilm. (TIF 1096 kb) [file 12866_2018_1224_MOESM3_ESM.tif]

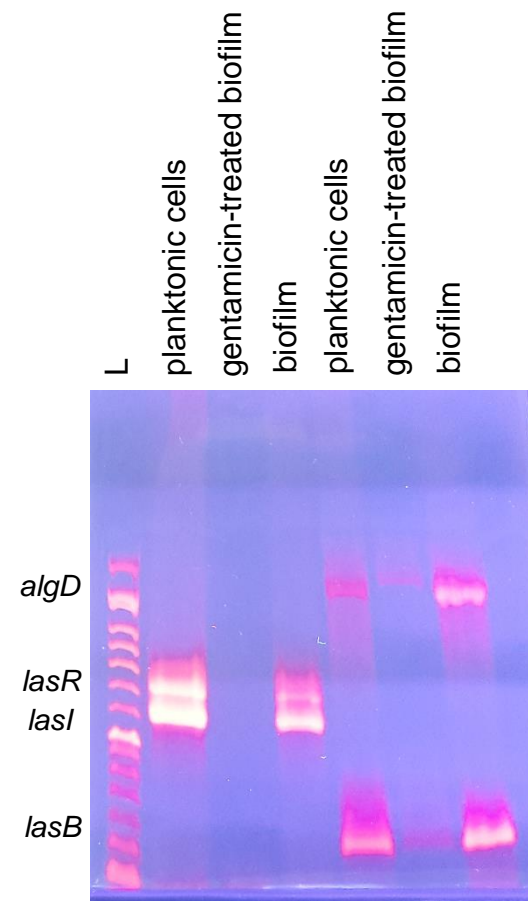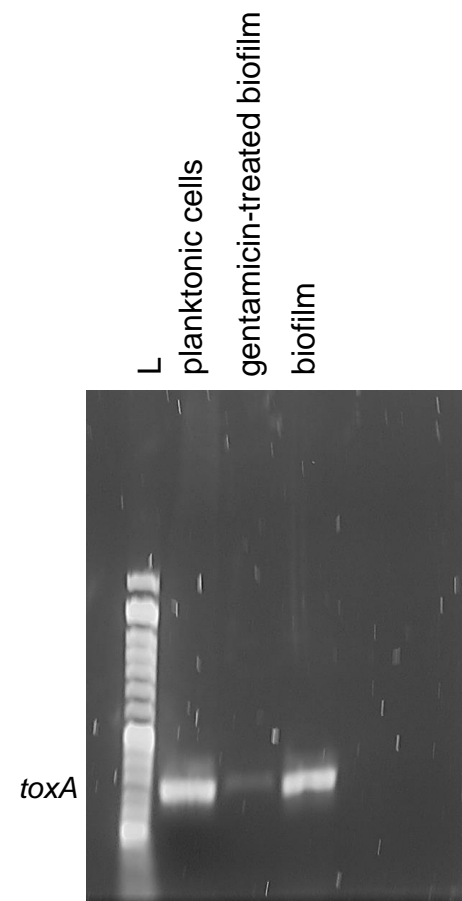

Additional figure 4

Supplement: Supplementary file 4 — Analysis of biofilm-related genes. BLI-Pseudomonas quorum sensing genes (lasR/lasI, lasB, toxA, algD) in a 24 h-old biofilm formed on ETT pieces and in the planktonic cells are shown. Lanes show the bands of each gene, according to the length of fragments expected, detected by gel analysis: lasR: 725 bp; lasI: 605 bp; lasB 300 bp, toxA 352 bp and algD 1310 bp (L = ladder). Gentamicin-treated biofilm served as control. (PDF 50 kb) [file 12866_2018_1224_MOESM4_ESM.pdf]
